# Co-expression of a SARP Family Activator ChlF2 and a Type II Thioesterase ChlK Led to High Production of Chlorothricin in *Streptomyces antibioticus* DSM 40725

**DOI:** 10.3389/fbioe.2020.01013

**Published:** 2020-08-21

**Authors:** Yue Li, Jihui Zhang, Jiazhen Zheng, Hanye Guan, Wen Liu, Huarong Tan

**Affiliations:** ^1^State Key Laboratory of Microbial Resources, Institute of Microbiology, Chinese Academy of Sciences, Beijing, China; ^2^University of Chinese Academy of Sciences, Beijing, China; ^3^State Key Laboratory of Bioorganic and Natural Products Chemistry, Shanghai Institute of Organic Chemistry, Chinese Academy of Sciences, Shanghai, China

**Keywords:** *Streptomyces antibioticus*, gene regulation, chlorothricin, high production, halogenase

## Abstract

Chlorothricin (CHL), produced by *Streptomyces antibioticus* DSM 40725 (wild-type strain, WT), belongs to a growing family of spirotetronate antibiotics that have biological activities inhibiting pyruvate carboxylase and malate dehydrogenase. ChlF2, a cluster-situated SARP regulator, can activate the transcription of *chlJ*, *chlC3*, *chlC6*, *chlE1*, *chlM*, and *chlL* to control CHL biosynthesis. Co-expression of *chlF2* and *chlK* encoding type II thioesterase in WT strain under the control of P*_*kan*_* led to high production of chlorothricin by 840% in comparison with that of WT. Since the inhibitory activity of CHL against several Gram-positive bacteria is higher than des-CHL, combinatorial strategies were applied to promote the conversion of des-CHL to CHL. Over-expression of *chlB4*, encoding a halogenase, combining with the supplementation of sodium chloride led to further 41% increase of CHL production compared to that of F2OE, a *chlF2* over-expression strain. These findings provide new insights into the fine-tuned regulation of spirotetronate family of antibiotics and the construction of high-yield engineered strains.

## Introduction

Streptomycetes are the most abundant source of antibiotics widely applied in clinical treatments, animal husbandry, and plant crop protection (Chen et al., 2008; Guo et al., 2010; Zhang et al., 2016). High-producing strains are demanded to meet the needs of industrial production (Li D. et al., 2019; Zheng et al., 2019). It is common that antibiotic biosynthesis is specified by the corresponding gene cluster, usually including pathway-specific regulatory genes (Liu et al., 2013; Li and Tan, 2017; Guan et al., 2019). In recent years, spirotetronate polyketides have been defined as a new family of microbial metabolites with potent antitumor properties or antibacterial activity, of which chlorothricin (CHL) was discovered as the first member of the spirotetronate family in 1969 (Lacoske and Theodorakis, 2015). Of the spirotetronate/spirotetramate natural products, members of this family usually possess a characteristic pentacyclic aglycone comprising a trans-decalin system and a tetronate or tetramate spiro-conjugate, e.g., kijanimicin, pyrrolosporin, tetrocarcin, lobophorin, versipelostatin, nomimicin, and abyssomicin (Igarashi et al., 2012; Li S. et al., 2013; Lacoske and Theodorakis, 2015; Tian et al., 2015). For instance, abyssomicin C and tetrocarcin A exhibit significant activity against antibiotic-resistant *Staphylococcus aureus* and *Mycobacterium tuberculosis*, as well as for the treatment of various lymphomas and solid tumors (Braddock and Theodorakis, 2019).

Chlorothricin possesses a pentacyclic aglycone that is conjugated with a modified methylsalicylic acid (MSA) moiety through a disaccharide linkage (Supplementary Figure S1) and shows good anti-inflammation activity, anti-cholesterol activity. Both CHL aglycone (chlorothricolide) and MSA are polyketide products assembled by iterative type I polyketide synthases, while MSA needs to be further functionalized by C2-O-methylation and C5-chlorination through ChlB5 and ChlB4, respectively (Yi et al., 2019). Disruption of *chlB4* and *chlB5* led to the accumulation of deschloro-chlorothricin (des-CHL) and desmethylsalicyl-CHL, respectively (Yi et al., 2019). The antibacterial activity is highly dependent on the glycosylation of chlorothricolide and halogenation of MSA. The glycosylation of chlorothricolide is also crucial for stability (Jia et al., 2006; Shao et al., 2006; Ding et al., 2010).

The regulation of biosynthesis of the secondary metabolites usually involves a complex regulatory network that responds to environmental or nutritional factors, signaling molecules, even the antibiotics themselves or their intermediates (Liu et al., 2013). The biosynthesis of most antibiotics is typically catalyzed by a series of enzymes that are encoded by structural genes and modulated by relevant pathway-specific regulators (Liu et al., 2013). Our previous work showed that ChlF1 is an essential activator and directly activates the transcription of the *chlJ*, which encodes an acyl-CoA carboxyl transferase involved in the CHL biosynthesis, while represses the transcription of *chlF1*, *chlK*, and *chlG* (Li Y. et al., 2016). We have revealed that the glycosylated end product CHL and its intermediates can act as signaling molecules of the regulator ChlF1. Also, the antibacterial activity of these molecules is correlated with their binding affinity to ChlF1 (Li Y. et al., 2016). But the role of another cluster-situated regulator (CSR) ChlF2 has not been reported up to now.

ChlF2 belongs to the family of *Streptomyces* antibiotic regulatory proteins (SARPs), which have a typical N-terminal OmpR-type DNA-binding domain and an additional bacterial transcriptional activation domain (Tanaka et al., 2007). Representative examples of SARPs include ActII-ORF4 and RedD from *Streptomyces coelicolor* A3 (2), which have function in the regulation of actinorhodin and undecylprodigiosin biosynthesis, respectively. A SARP-type regulator TylS is essential for tylosin production and controls the expression of another global activator gene *tylR* (Bate et al., 2002). Heterologous expression of SARP-type regulator gene *papR2* from *Streptomyces pristinaespiralis* in *Streptomyces lividans* resulted in the activation of the silent undecylprodigiosin (Red) gene cluster (Krause et al., 2020).

In this article, we demonstrate that ChlF2 is an essential regulator for CHL biosynthesis. It positively controls the transcription of *chlJ*, *chlC3*, *chlC6*, *chlE1*, *chlM*, and *chlL*. Over-expression of *chlF2* and its co-transcribed type II thioesterase coding gene *chlK* effectively increased CHL production by 840% in comparison to WT production levels. We provide some evidences that des-CHL and CHL have inhibitory activity against cancer cells. Meanwhile, CHL exhibits higher biological activities than des-CHL against several Gram-positive bacteria. Since ChlB4 is responsible for the C5-chlorination of MSA, over-expression of *chlB4* was performed to promote the production of CHL, which is further enhanced with the addition of sodium chloride. These findings provide new insights into the fine-tuned regulation of the biosynthesis of spirotetronate antibiotics and lay foundation for constructing high-yield engineered strains.

## Materials and Methods

### Media and Strain Growth Conditions

*Streptomyces* were grown on MS agar (2% soybean flour, 2% mannitol, 2% agar) for spore preparation and conjugal transfer, and in liquid YEME medium (0.3% yeast extract, 0.5% tryptone, 0.3% malt extract, 20% sucrose, 5 mM MgCl_2_, 1% glucose, 0.5% glycine) for total DNA isolation. *Streptomyces coelicolor* M1146 and its derivatives were cultured on AS-1 agar (0.1% yeast extract, 0.02% L-alanine, 0.02% L-arginine, 0.02% L-asparagine, 0.5% soluble starch, 0.25% NaCl, and 1% Na_2_SO_4_) for phenotypical analysis. *Escherichia coli* JM109 was used as a general host for propagating plasmids at 37°C. *E. coli* ET12567/pUZ8002 was used for *E. coli–Streptomyces* conjugal transfer. The temperature-sensitive shuttle plasmid pKC1139 was used to construct recombinant plasmids for gene disruption and over-expression. The integrative plasmids pSET152 and pIJ10500, which can integrate into the chromosome by site-specific recombination at the phage ΦC31 or ΦBT1 attachment site (*attB*), respectively, were used for gene complementation, over-expression, or *gusA* reporter system (Supplementary Table S1). When necessary, antibiotics were used at the following final concentrations: 100 μg/ml of apramycin, ampicillin, kanamycin, or hygromycin and 50 μg/ml of chloramphenicol in LB for *E. coli*; 25 μg/ml of nalidixic acid or apramycin, 50 μg/ml of kanamycin, and 12.5 μg/ml of hygromycin in MS for *Streptomyces antibioticus*; 25 μg/ml of nalidixic acid and 50 μg/ml of apramycin, kanamycin, or hygromycin in MS for *Streptomyces coelicolor*.

### Plasmids and Strains Construction

All the plasmids, strains, and primers used in this work are listed in Supplementary Tables S1–S3, respectively, and the detailed procedures for plasmids and strains construction are described in Supplementary Material.

### Fermentation, Isolation, and Analysis of Chlorothricin

The spore suspension was inoculated into a 250-ml flask containing 50 ml YEME medium, incubated for 48 h in a rotary shaker at 220 rpm and 28°C as the seed culture. The seed culture was inoculated into fermentation medium (2% cold pressed soybean flour, 2% mannitol, 0.2% CaCO_3_, pH 6.8) for CHL and des-CHL production. Cultures of *S. antibioticus* grown at 28°C for 7 days were harvested and extracted with methanol. The samples were analyzed on Agilent 1100 HPLC (Zorbax, SB-C18, 4.6 × 250 mm, 5 μm) at detection wavelength 222 nm. des-CHL and CHL were identified by LC-MS as previously described (Li Y. et al., 2016).

### RNA Isolation and Quantitative Real-Time PCR

This experiment was performed as previously described (Li Y. et al., 2016). Total RNA was isolated from cultures of *S. antibioticus* grown at several time points (48, 72, and 96 h); 500 ng of pure DNA-free RNA was used to prepare cDNA, and each 20 μl of PCR reaction mixture contained 10 μl of 2× SuperReal PreMix, 6 pmol of each primer, and 1 μl of threefold diluted cDNA (1000-fold diluted for amplification of 16S rRNA as internal control). The primers used for qRT-PCR were listed in Supplementary Table S3. RT-qPCR was performed in a Rotor-Gene Q with FastFire qPCR PreMix (SYBR Green) kit according to the manufacturer’s instructions.

### Bioassay of Des-CHL and CHL

To detect the biological activities of des-CHL and CHL against *Bacillus subtilis* CGMCC 1.1630 (*B. subtilis*) and *Staphylococcus aureus* CGMCC 1.89 (*S. aureus*), the purified des-CHL and CHL dissolved in 30 μl methanol were added into the holes with 0.8 cm diameter in LB agar medium containing 1% (v/v) *B. subtilis or S. aureus* culture, respectively. The plate was incubated at 37°C for 8 h, and the antibacterial activity was estimated by measuring the diameter of the inhibition zones.

### Determination of Minimum Inhibitory Concentration and 50% Inhibitory Concentration

To determine the minimum inhibitory concentrations (MICs) of des-CHL and CHL on bacteria, antibacterial activity was measured according to the method described previously (Lu et al., 2015). Purified CHL and des-CHL were dissolved in DMSO to form a stock solution (100 mg/ml) and serially diluted with LB prior to mixing with indicator strains, which were pre-incubated in LB on a rotary shaker at 37°C for overnight. Assays for determining MIC were performed on 96-well microtiter plates containing diluted compounds, indicator strains (0.5%) and 0.5% DMSO. The growth of indicator strains was measured after 12 h of incubation for *Staphylococcus epidermidis* ATCC 35984 (*S. epidermidis*), *S. aureus, B. subtilis*, and *Bacillus cereus* CGMCC 1.1626 (*B. cereus*), and 18 h for *Streptococcus pneumoniae* 010 (*S. pneumoniae*) and *Streptococcus pyogenes* #2 (*S. pyogenes*) on a microplate reader (Epoch, Biotech) at wavelength of 600 nm (Supplementary Table S2). Strains grown in LB medium containing 0.5% DMSO without test compounds were used as positive controls, and LB medium containing 0.5% DMSO was used as blank. All tests were carried out in triplicate. The 50% inhibitory concentration (IC_50_) against cancer cell lines was performed as described previously (Xu et al., 2017), in which human lung carcinoma cell line A549, human lung adenocarcinoma cell line Calu-3, human hepatocellular carcinoma cell line HepG2, and human breast adenocarcinoma cell line MCF-7 were used.

## Results

### A SARP Family Activator ChlF2 Positively Regulates CHL Biosynthesis

*Streptomyces* antibiotic regulatory protein family regulators are widespread in Streptomycetes (Krause et al., 2020). The deduced product of *chlF2* of *S. antibioticus* is a protein of 262 aa, containing a typical DNA binding domain at the N-terminus and two other functional domains at the C-terminus: a BTA (bacterial transcriptional activator) domain and a tetratricopeptide repeat (TPR) domain usually mediating protein-protein interactions and assembly of multi-protein complex (Supplementary Figure S2A). ChlF2 was deduced to be a SARP-type regulator, which shows 68% amino acid sequence identity with PlaR1 involved in phenalinolactone biosynthesis of *Streptomyces* sp. TÜ6071 (Myronovskyi et al., 2011), 59% identity with PyrF2 involved in pyrroindomycin biosynthesis of *Streptomyces rugosporus* (Wu et al., 2012), and 55% and 53% identity with NanR2 and NanR1 involved in nanchangmycin biosynthesis of *Streptomyces nanchangensis NS3226* (Sun et al., 2003), respectively (Supplementary Figure S2B).

In order to determine the role of ChlF2 in CHL biosynthesis, a *chlF2* disruption mutant (ΔchlF2) was constructed via homologous recombination. In ΔchlF2, a 708 bp internal fragment of *chlF2* was replaced by a kanamycin resistance gene (*kan)* in its parent strain *S. antibioticus* DSM 40725. Southern blot experiment was carried out to confirm the correctness of ΔchlF2 mutant. Genomic DNAs of seven conjugants and WT were digested with *Nco*I, and 1 kb DNA fragment containing *kan* from pUC119:*neo* was used as probe. As expected, all these preliminarily selected transformants exhibited positive signals at 2363 bp and 794 bp, whereas WT strain did not exhibit signal due to the absence of *kan*, indicating that the resulting disruption strains were correct (Supplementary Figures S3A,B).

**FIGURE 1:**
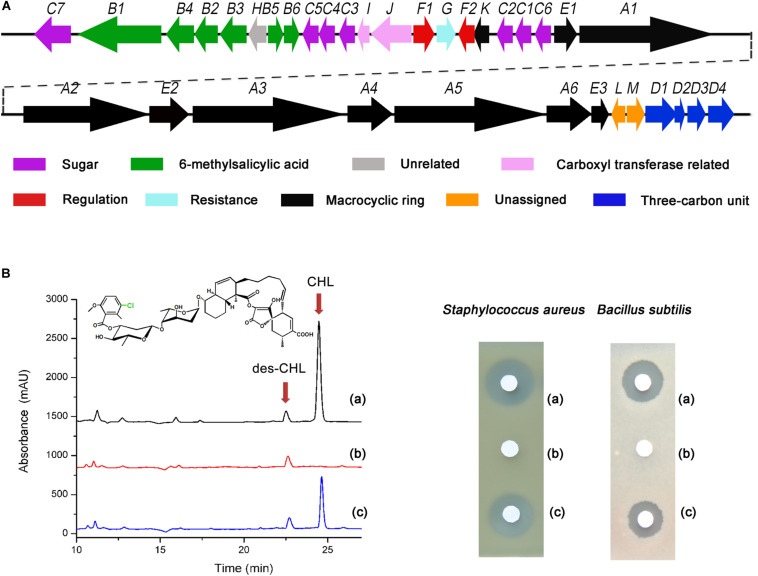
Effect of *chlF2* disruption on CHL production. **(A)** Genetic organization of the *chl* cluster. Each *arrow* indicates a separate ORF and orientation of the transcription. **(B)** HPLC analysis and bioassays of CHL produced by WT and its derivatives. (a) Wild-type *S. antibioticus* DSM 40725; (b) ΔchlF2, *chlF2* disruption mutant; (c) ΔchlF2/pSET152:P*_*chlK*_*-*chlK-chlF2*, *chlF2* complementary strain. des-CHL indicates deschloro-chlorothricin, CHL indicates chlorothricin. The chemical structure of CHL is presented, and the position of Cl is indicated with green color.

The fermentation of ΔchlF2 and WT strain was carried out to compare the change of CHL production. HPLC analysis revealed that a characteristic peak corresponding to CHL standard in the extract of WT disappeared in the ΔchlF2 mutant (Figures 1A,B). Also, an inhibition zone was observed with the fractions from WT but not with that from ΔchlF2 in the bioassays against *S. aureus* and *B. subtilis*, suggesting that CHL biosynthesis was abolished in ΔchlF2. To verify whether this was due to *chlF2* disruption, the *chlF2* complementary strain ΔchlF2/pSET152:P*_*up*__*chl*__*F*__2_-chlF2*, in which the transcription of *chlF2* was driven by a deduced promoter sequence (a short stretch of upstream *chlF2* coding sequence), was constructed based on the integrative plasmid pSET152. Unexpectedly, there was no peak corresponding to CHL detected in the fermentation broth of ΔchlF2/pSET152:P*_*upchl*__*F*__2_-chlF2* (data not shown). Further experiment demonstrated that CHL production could not be restored unless *chlF2* and its adjacent gene *chlK* were co-expressed under the control of the native promoter P*_*chlK*_*, as shown by HPLC analysis and the bioassays against *S. aureus* and *B. subtilis* (Figures 1A,B), implying that *chlF2* transcription may depend on the promoter of the adjacent gene *chlK.* The above results indicated that ChlF2 is an activator involved in CHL biosynthesis.

In order to confirm whether *chlK* and *chlF2* are co-transcribed, RT-PCR experiment was carried out. Primer pairs PF2-F/PK-R and B16S-F/B16S-R were used to confirm the transcriptional profile of *chlKF2* and 16S rRNA coding gene. The results showed that the expected sizes of PCR fragments (429 bp for 16s rRNA coding gene and 1398 bp for *chlKF2*) were observed in accordance with the positive control (genome DNA of WT as template), but not visible in the negative control (RNA without reverse transcription as template), indicating that *chlK* and *chlF2* were located in the same operon (Supplementary Figures S4A,B). Overall, these results confirmed that *chlF2* is an indispensable activator gene for CHL production.

### ChlF2 Binds to Several Promoter Regions

**FIGURE 2:**
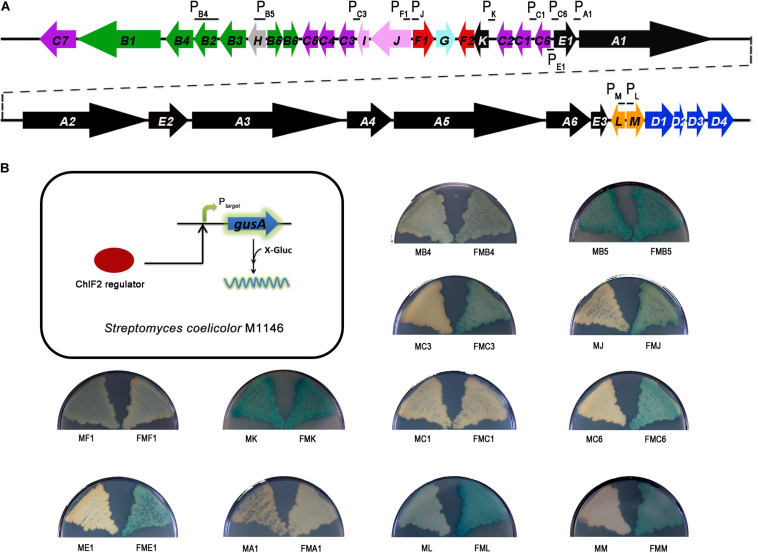
In vivo determination of the interaction between ChlF2 and its targets. **(A)** Promoter regions selected for detecting the binding activity of ChlF2 were indicated by short black lines in *chl* cluster. **(B)** Schematic diagram of *gusA* transcriptional fusion assays and chromogenic assays of *S. coelicolor* M1146 derivatives. MB4, MB5, MC3, MJ, MF1, MK, MC1, MC6, ME1, MA1, ML, and MM strains were used as controls. FMB4, FMB5, FMC3, FMJ, FMF1, FMK, FMC1, FMC6, FME1, FMA1, FML, and FMM strains were used to identify the binding regions of ChlF2.

To find the direct targets of ChlF2 in the *chl* cluster, we first tried to purify ChlF2 prior to the electrophoretic mobility shift assay (EMSA), but unfortunately, we could not get the soluble protein after over-expression of *chlF2 in vitro*. Therefore, the *in vivo* reporting system of *gusA* was taken into account (Sherwood and Bibb, 2013). Several promoters (P*_*chlB*__4_*, P*_*chlB*__5_*, P*_*chlC*__3_*, P*_*chlJ*_*, P*_*chlF*__1_*, P*_*chlK*_*, P*_*chlC*__1_*, P*_*chlC*__6_*, P*_*chlE*__1_*, P*_*chlA*__1_*, P*_*chlL*_*, and P*_*chlM*_*) were chosen and individually inserted upstream of the *gusA* coding region (encoding β-glucuronidase, GUS) (Figure 2A). The *gusA*-fused plasmids were respectively introduced into *S. coelicolor* M1146 to generate recombinant strains MB4 (*S. coelicolor* M1146/pIJ10500:P*_*chlB*__4_*-*gusA*), MB5 (M1146/pIJ10500:P*_*chlB*__5_*-*gusA*), MC3 (M1146/pIJ10500:P*_*chlC*__3_*-*gusA*), MJ (M1146/pIJ10500:P*_*chlJ*_*-*gusA*), MF1 (M1146/pIJ10500:P*_*chlF*__1_*-*gusA*), MK (M1146/pIJ10500:P*_*chlK*_*-*gusA*), MC1 (M1146/pIJ10500:P*_*chlC*__1_*-*gusA*), MC6 (M1146/pIJ10500:P*_*chlC*__6_*-*gusA*), ME1 (M1146/pIJ10500:P*_*chlE*__1_*-*gusA*), MA1 (M1146/pIJ10500:P*_*chlA*__1_*-*gusA*), ML (M1146/pIJ10500:P*_*chlL*_*-*gusA*), and MM (M1146/pIJ10500:P*_*chlM*_*-*gusA*), and GUS activity (for hydrolyzing 5-bromo-4-chloro-3-indolyl-β-d-glucuronide to the blue product 5,5′-dibromo-4,4′-dichloro-indigo) was subsequently observed via color change. A *chlF2* over-expression plasmid pKC1139*-P_*kan*_-kan-chlF2* was integrated into the φC31 *attB* site of the above-mentioned constructed recombinant strains to get the corresponding reporter strains FMB4 (*S. coelicolor* M1146/pIJ10500:P*_*chlB*__4_*-*gusA* and pKC1139*-P_*kan*_-kan-chlF2*), FMB5 (M1146/pIJ10500:P*_*chlB*__5_*-*gusA* and pKC1139*-P_*kan*_-kan-chlF2*), FMC3 (M1146/pIJ10500:P*_*chlC*__3_*-*gusA* and pKC1139*-P_*kan*_-kan-chlF2*), FMJ (M1146/pIJ10500:P*_*chlJ*_*-*gusA* and pKC1139*-P_*kan*_-kan-chlF2*), FMF1 (M1146/pIJ10500:P*_*chlF*__1_*-*gusA* and pKC1139*-P_*kan*_-kan-chlF2*), FMK (M1146/pIJ10500:P*_*chlK*_*-*gusA* and pKC1139*-P_*kan*_-kan-chlF2*), FMC1 (M1146/pIJ10500:P*_*chlC*__1_*-*gusA* and pKC1139*-P_*kan*_-kan-chlF2*), FMC6 (M1146/pIJ10500:P*_*chlC*__6_*-*gusA* and pKC1139*-P_*kan*_-kan-chlF2*), FME1 (M1146/pIJ10500:P*_*chlE*__1_*-*gusA* and pKC1139*-P_*kan*_-kan-chlF2*), FMA1 (M1146/pIJ10500:P*_*chlA*__1_*-*gusA* and pKC1139*-P_*kan*_-kan-chlF2*), FML (M1146/pIJ10500:P*_*chlL*_*-*gusA* and pKC1139*-P_*kan*_-kan-chlF2*), and FMM (M1146/pIJ10500:P*_*chlM*_*-*gusA* and pKC1139*-P_*kan*_-kan-chlF2*) accordingly. If ChlF2 could bind to the target genes, the expression of *gusA* would be activated to show blue color. The results showed that GUS activity of FMC3, FMJ, FMC6, FME1, FML, and FMM strains were obviously observed, revealing that the promoters of *chlC3*, *chlJ, chlC6, chlE1, chlL*, and *chlM* are probably the targets of ChlF2 (Figure 2B). It was noteworthy that GUS activity was detected in strains MB5, MB4, MF1, and MK, in which ChlF2 is absent, implying that *chlB5, chlB4, chlF1*, and *chlK* were constitutively expressed but not regulated by ChlF2. Neither FMC1 nor FMA1 activity was detectable under both conditions, indicating that ChlF2 could not bind to the promoters of *chlC1* and *chlA1* (Figure 2B).

### ChlF2 Activates the Transcription of Several Structural Genes

**FIGURE 3:**
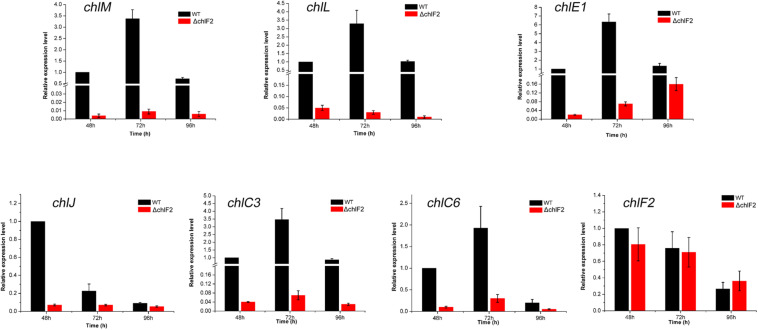
RT-qPCR transcriptional analysis of selected *chl* genes in WT and ΔchlF2. RNA samples were isolated from fermentation cultures after incubation for 48, 72, and 96 h. The data are presented as the averages of three independent experiments conducted in triplicate. Error bars show standard deviations.

To determine the effect of *chlF2* disruption on its target genes, quantitative real-time PCR (RT-qPCR) analysis was performed to assess the transcriptional profiles of target genes *chlM*, *chlL*, *chlE1*, *chlJ*, *chlC3*, *chlC6*, and non-target gene *chlF2* as a negative control. Total RNA was prepared from the cultures of WT and ΔchlF2 at different time points (48 h, 72 h, 96 h). The results showed that the transcripts of *chlM*, *chlL*, *chlE1, chlC3, chlJ*, and *chlC6* were significantly decreased in ΔchlF2 at each time point in comparison with those in WT strain (Figure 3), revealing that ChlF2 can activate the transcription of these genes directly. The transcription of non-target gene *chlF2* was not affected (Figure 3). These data indicated that ChlF2 positively regulates its target genes.

### Over-Expression of *chlF2* Increased the Production of CHL and Des-CHL

**FIGURE 4:**
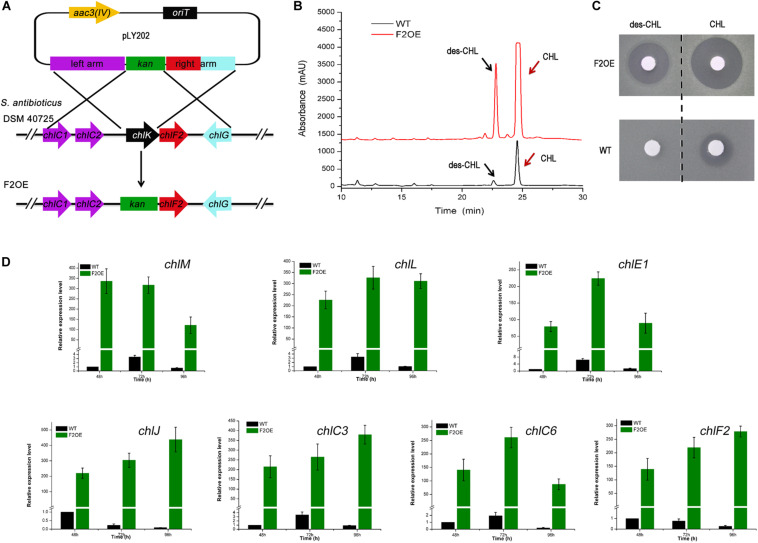
Metabolite analysis of WT and F2OE. **(A)** Construction of *chlF2* over-expression engineered strain (*S. antibioticus* F2OE) via homologous recombination. *chlK* was replaced by *kan* and its promoter P*_*kan*_* on the chromosome. **(B)** HPLC analysis of the extracts of WT and *S. antibioticus* F2OE. des-CHL indicates deschloro-chlorothricin, CHL indicates chlorothricin. **(C)** Bioassays of the extracts of WT and *S. antibioticus* F2OE against *B. subtilis*. **(D)** RT-qPCR transcriptional analysis of selected *chl* genes in WT and F2OE. The data are presented as the averages of three independent experiments conducted in triplicate. Error bars show standard deviations.

ChlF2 is an important activator of CHL biosynthesis, and *chlF2* complementary experiments showed that *chlK* and *chlF2* are located in the same transcriptional unit under the control of P*_*chlK*_* (Figure 1A and Supplementary Figure S4). Previous studies on the role of ChlK showed that in-frame deletion of *chlK* led to partial decrease of CHL (Li Y. et al., 2016), whereas remarkable enhancement was observed in mutant F2OE, in which *chlK* was replaced by a resistance gene *kan* and its promoter P*_*kan*_* via homologous recombination. Actually *chlF2* and *kan* are co-transcribed under the control of strong promoter P*_*kan*_* (Figures 4A,B). Bioassays were performed against *B. subtilis* with the fractions corresponding to CHL and des-CHL from WT and F2OE based on HPLC analysis. Inhibition zones with the fractions of des-CHL and CHL from F2OE were much bigger than those from WT, which were consistent with the HPLC data (Figure 4C). These results strongly supported that ChlF2 is a crucial activator for CHL biosynthesis, and ChlK exerts some functions but is not decisive. The transcriptional levels of all six target genes in F2OE remarkably increased at each time point compared with those in WT. A more than 100-fold transcriptional enhancement of the non-target gene *chlF2* in F2OE was likely attributed to the exogenous insertion of constitutive promoter P*_*kan*_* (Figure 4D).

**FIGURE 5:**
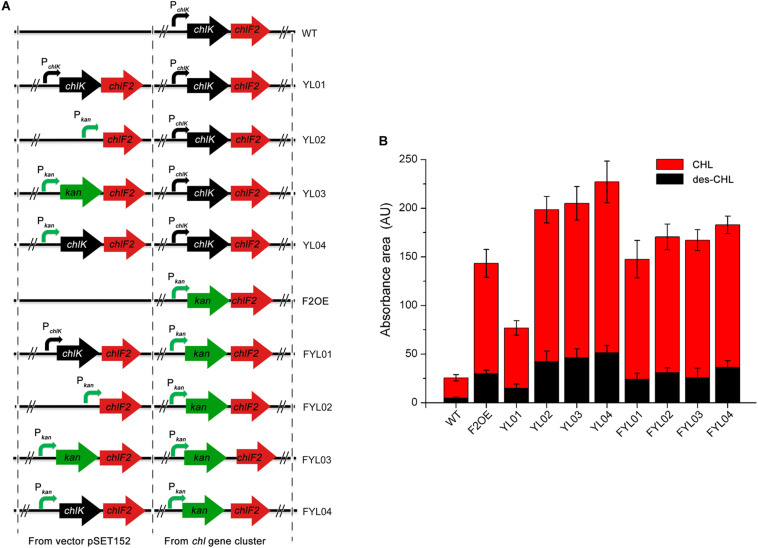
Comparison of antibiotics production between WT and its recombinant over-expression strains. **(A)** The constructions of *chlF2* over-expression strains. **(B)** Absorbance area of des-CHL and CHL detected by HPLC in WT and its derivatives. CHL, chlorothricin; des-CHL, deschloro-chlorothricin.

To enhance CHL production effectively, several strategies toward *chlF2* and *chlK* were attempted to understand their role. Firstly, the integrative plasmids pSET152:P*_*chlK*_-chlK-chlF2*, pSET152:P*_*kan*_-chlF2*, pSET152:P*_*kan*_-kan-chlF2*, and pSET152:P*_*kan*_-chlK-chlF2* were constructed and introduced into WT, respectively. The resulting recombinant strains were named as YL01, YL02, YL03, and YL04 accordingly (Figure 5A). After fermentation and HPLC analysis, in comparison with WT, the production of CHL and des-CHL was increased by 300% and 310% in YL01, and 750% and 900% in YL02, respectively (Figure 5B). The CHL production in YL03 and YL04 was increased by 760% and 840% compared with WT, respectively (Figure 5B). Furthermore, des-CHL was significantly increased by 980% and 1100%, respectively. These results revealed the importance of promoter P*_*kan*_* for *chlF2* and *chlK* expression, and which in turn led to high-production of CHL and des-CHL.

Since F2OE exhibited much higher production of CHL than WT, it was consequently used as an alternative host strain to further improve the antibiotics production. The following four integrative plasmids pSET152:P*_*chlK*_-chlK-chlF2*, pSET152:P*_*kan*_-chlF2*, pSET152:P*_*kan*_-kan-chlF2*, and pSET152:P*_*kan*_-chlK-chlF2* were separately introduced into F2OE to generate strains FYL01, FYL02, FYL03 and FYL04 (Figure 5A). HPLC analysis indicated that a range of 590%∼700% improvement of CHL production was observed in these strains compared to WT strain, even though FYL02, FYL03, and FYL04 did not exhibit higher production of des-CHL and CHL than YL02, YL03, and YL04 (Figure 5B), implying strong promoter and cellular tolerance are crucial elements. Thus, YL04 was selected as the high-yield producing strains of CHL and des-CHL for further studies.

### Bioassays of CHL and Des-CHL Against Bacteria and Tumor Cells

**FIGURE 6:**
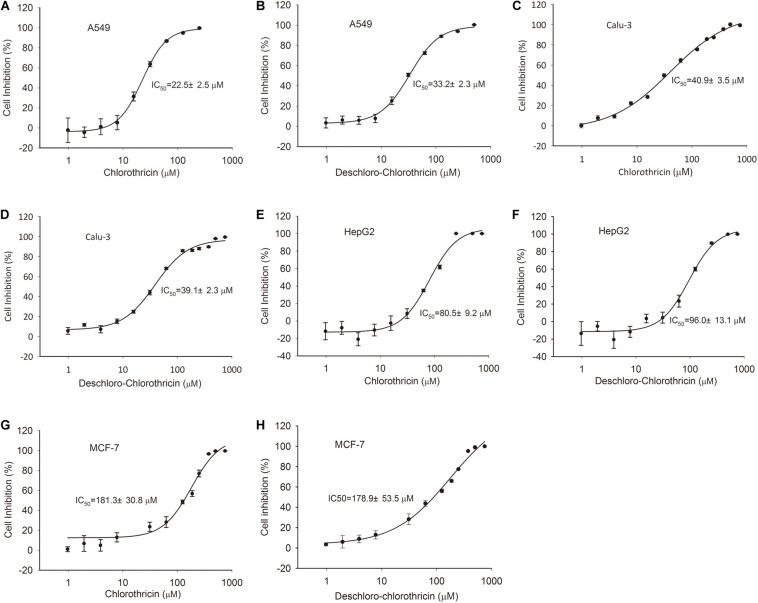
Cytotoxic assays of CHL and des-CHL. Cytotoxic assays of CHL against A549 **(A)**, Calu-3 **(C)**, HepG2 **(E)**, and MCF-7 **(G)**. Cytotoxic assays of des-CHL against A549 **(B)**, Calu-3 **(D)**, HepG2 **(F)**, and MCF-7 **(H)**. CHL, chlorothricin; des-CHL, deschloro-chlorothricin. A549 indicates human lung carcinoma cell line, Calu-3 indicates human lung adenocarcinoma cell line, HepG2 indicates human hepatocellular carcinoma cell line, MCF-7 indicates human breast adenocarcinoma cell line. IC50 (50% inhibitory concentration) values were labeled. The error bars indicated the standard deviations (SD) of three independent experiments.

Chlorothricin and des-CHL are two major spirotetronate components. To determine their bioactivities, CHL and des-CHL were purified from YL04 high-producing strain, and their antibacterial and anticancer activities were evaluated. MIC values were determined against several Gram-positive bacteria, which ranged from 31.25 to 100.00 μg/ml. CHL showed improved antibacterial activity in comparison with des-CHL against *B. subtilis, B. cereus* and *S. aureus* (Table 1). For instance, MIC of CHL was 31.25 μg/ml against the above three indicator strains, while MIC of des-CHL was 62.5 μg/ml against *B. subtilis* and *B. cereus* (Table 1). CHL and des-CHL showed less than 80% inhibitory activity at the maximum tested concentration 100 μg/ml against *S. epidermidis*, *S. pyogenes*, and *S. pneumoniae*. In addition, we could not detect any biological activity against *P. aeruginosa* PA14, *A. longipes* CGMCC 3.2946, and *C. albicans* CGMCC 2.4159 (Table 1). Meanwhile, the CHL and des-CHL showed inhibitory activity against A549 (non-small-cell lung cancer) and Calu-3 (human lung adenocarcinoma cancer), HepG2 (liver cancer), and MCF-7 (breast cancer) with IC_50_ ranging from 22.5 μM to 181.3 μM (Figure 6). Overall, both compounds exhibited wide antibacterial or anticancer cell line spectrum, indicating their potential for drug development.

**TABLE 1:** Antimicrobial activities of des-CHL and CHL.

**Indicator strains**	**MIC (μg/ml)**	
	**CHL**	**des-CHL**
Gram-positive bacteria		
*Bacillus subtilis* CGMCC 1.1630	31.25	62.5
*Bacillus cereus* CGMCC 1.1626	31.25	62.5
*Staphylococcus aureus* CGMCC 1.89	31.25	>100
*Staphylococcus epidermidis* ATCC 35984	100	>100
*Streptococcus pyogenes* #2	>100	>100
*Streptococcus pneumoniae* 010	>100	>100
Gram-negative bacteria		
*Pseudomonas aeruginosa* PA14	No	No
Fungus		
*Alternaria longipes* CGMCC 3.2946	No	No
*Candida albicans* CGMCC 2.4159	No	No

### Combination of *chlB4* Over-Expression and Medium Optimization Promotes the Conversion of Des-CHL to CHL

**FIGURE 7:**
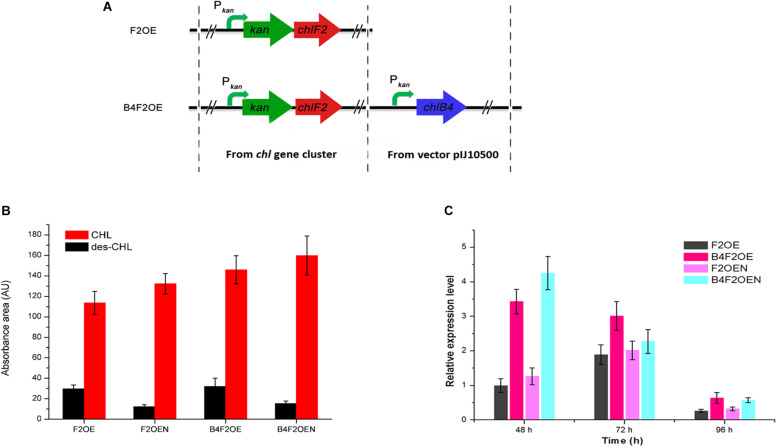
Metabolites analysis of WT and its recombinant strains. **(A)** The construction of B4F2OE strain. **(B)** HPLC analysis of the extracts of WT and its recombinant strains. des-CHL indicates deschloro-chlorothricin, CHL indicates chlorothricin. **(C)** RT-qPCR analysis of *chlB4* in F2OE, F2OEN, B4F2OE and B4F2OEN. WT, *S. antibioticus* DSM 40725; WTN, WT with the addition of 0.05% NaCl when fermentation; F2OE, *chlF2* over-expression strain; F2OEN, F2OE with the addition of 0.05% NaCl when fermentation; B4F2OE, halogenase coding gene *chlB4* and *chlF2* co-expression strain, B4F2OEN, B4F2OE with the addition of 0.05% NaCl when fermentation.

As aforementioned, over-expression of *chlK-chlF2* or *kan-chlF2* under the control of P*_*kan*_* led to accumulation of des-CHL. Because CHL exhibits higher activity than des-CHL against *B. cereus, B. subtilis*, and *S. aureus*, it is necessary to promote the conversion of des-CHL to CHL. *chlB4* encoding halogenase is a key cluster-situated gene that responsible for the halogenation of MSA to form CHL. We speculated that the possible insufficient expression of halogenase ChlB4 or shortage of chloride ions in the medium could be a limiting factor for the accumulation of des-CHL. Thus, in order to further improve the production of CHL, the *chlB4* was first over-expressed under the control of P*_*kan*_* in F2OE to generate B4F2OE strain (Figure 7A). The result showed that both des-CHL and CHL of B4F2OE increased 7% and 29% compared with that of F2OE, respectively (Figure 7B). When optimal concentration of 0.05% NaCl as the chloride reservoir was added into the medium before fermentation, des-CHL production in B4F2OEN was markedly decreased about 52% in comparison to that in F2OE, while CHL increased 41% (Figure 7B). These results indicated that combination of the two strategies together can efficiently promote the conversion of des-CHL to CHL. To investigate the transcriptional level of *chlB4* in F2OE, F2OEN, B4F2OE, and B4F2OEN, RT-qPCR experiment showed that the transcripts of *chlB4* in F2OEN, B4F2OE, and B4F2OEN were all upregulated in varying degrees at different time points (Figure 7C).

## Discussion

*Streptomyces* antibiotic regulatory proteins (SARPs) are a specific family of paralogous proteins that are associated with antibiotic biosynthesis in many streptomycetes. SARPs are widespread in *Streptomyces* with a varying length (from less than 300 aa to more than 1000 aa) and have three major functional domains (a N-terminal DNA binding domain, a central AAA domain and a conserved C-terminal domain of unknown function) (Liu et al., 2013). As we know, the biosynthesis of antibiotics is often hierarchically controlled by multi-level regulators in *Streptomyces* (Li C. et al., 2019), some of which are cascade regulation. For example, SARP family regulator PolY can activate PolR, and the latter can regulate the transcription of the whole polyoxin biosynthetic gene cluster to control polyoxin biosynthesis in *Streptomyces cacaoi* subsp. *asoensis* (Li R. et al., 2009, 2010). Some regulators exercise synergistic regulation with other proteins, such as OvmZ, its interplay with OvmW can activate the transcription of *ovmOI-ovmH* operon to control oviedomycin production (Xu et al., 2017). Two pathway-specific regulatory genes *chlF1* and *chlF2* are included in *chl* gene cluster, so we are curious to know the relationship between ChlF1 and ChlF2. First of all, GUS activity assays demonstrated that the expression of *chlF1* was not regulated by ChlF2, while our previous EMSA experiments had illustrated that ChlF1 directly represses the transcription of *chlK* (Li Y. et al., 2016). Since *chlK* and *chlF2* are co-transcribed, *chlF2* was speculated to be regulated by ChlF1 through controlling the P*_*chlK*_* promoter. Subsequent RT-qPCR analysis confirmed that the transcription of *chlF2* was negatively controlled by ChlF1 (Supplementary Figure S5). However, unlike *chlF2*, the over-expression of *chlF1* driven by its own promoter did not lead to remarkable improvement of CHL production. Therefore, we speculated that the increase of CHL production in F2OE or YL04 is ascribed to the transcriptional enhancement of several structural genes activated by ChlF2 directly. Meanwhile, replacing P*_*chlK*_* with stronger promoter P*_*kan*_* could help relieve the repression of ChlF1 on ChlF2.

As aforementioned, co-expression of both *chlK* and *chlF2* or co-expression of both *kan* and *chlF2* significantly increased the yield of des-CHL and CHL. It is likely that the ChlK or KanP may act as ‘helper’ of ChlF2 or form heterodimer with ChlF2. To assess if ChlK and ChlF2 or KanP and ChlF2 interacted, bacterial two-hybrid system based on the reconstitution of activity of adenylate cyclase in *E. coli* BTH101 (*cya-*) was performed as described in the Supplementary Material. *chlK* and *kan* were respectively fused to the T25 domain of an adenylate cyclase coding gene to generate plasmids pKT25-*chlK* and pKT25-*kan*, and *chlF2* was fused to the T18 domain of the adenylate cyclase coding gene to generate plasmid pUT18-*chlF2*. Once the two proteins interacted, adenylate cyclase activity would be restored due to its T25 domain and T18 domain getting closer and then the strain would exhibit blue color on LB plate containing X-Gal (Supplementary Figure S6A). As a result, neither SZ-kanF2 nor SZ-KF2 strain exhibited blue color compared to the positive control SZ-zip, implying that ChlF2 and ChlK or ChlF2 and KanP don’t interact with each other *in vivo* (Supplementary Figure S6B). So it is unlikely that ChlK and KanP could facilitate CHL production via forming heterodimer with ChlF2. However, an alternative hypothesis about ChlK coordination with ChlF2 may be proposed based on the enzymatic activity. ChlK is a type II thioesterase and plays an ‘editor’ role to ensure the specific loading efficiency via hydrolyzing aberrantly loaded substrates. Our previous study showed that in-frame deletion of *chlK* resulted in declined CHL production, but not abolished completely (Li Y. et al., 2016), while over-expression of *chlK* along with *chlF2* in YL-series or FYL-series strains did improve CHL production. One of the underlying mechanisms about co-expression of ChlK and ChlF2 could be considered to enhance substrate loading efficiency and accuracy during the chlorothricolide or MSA (methylsalicylic acid) biosynthesis, and increase the enzyme activity of several target genes of ChlF2 which in turn enhanced the CHL production.

Since multiple components with different potency are usually co-produced as a mixture in the manufacture process of most microbial antibiotics. The selective enhancement of the desired components and reduction of the undesired components are always expected in metabolic engineering (Cropp et al., 2001). Thus, it is very important to confirm the crucial switch that controls the metabolic flux toward the desired antibiotics and then genetic manipulation of the corresponding genes. In WT, *chlB4* encoding halogenase is a key cluster-situated gene for catalyzing C5-chlorination of MSA. Its over-expression combined with the addition of NaCl facilitated the conversion of des-CHL to CHL. It can be perceived that more copies of *chlB4* and optimization of the medium component may lead to an even higher yield. Moreover, since a high proportion of CHL is accumulated intracellularly rather than being exported out of the cells, the overproduced CHL may cause feedback regulation as described in our previous study (Li Y. et al., 2016), enhancing the cellular tolerance in combination with the above strategies would be promising to further improve CHL yield.

Taken together, the clarification of the regulatory mechanisms paves a road to further optimize the corresponding genes for strain improvement beneficial for commercial application. The findings also provide basis for investigating the biosynthesis and regulation of other spirotetronate family of antibiotics.

## Data Availability Statement

All datasets generated for this study are included in the article/Supplementary Material.

## Author Contributions

YL carried out most of the experiments, analyzed the primary data, and wrote the draft manuscript. JHZ performed the anticancer experiment and revised the manuscript. JZZ and HG constructed part of recombinant plasmids and strains related to GUS assays. WL conceived the partial research work. HT conceived the whole research work and revised the manuscript. All authors read and approved the final manuscript.

## Conflict of Interest

The authors declare that the research was conducted in the absence of any commercial or financial relationships that could be construed as a potential conflict of interest.
