# Supplementary material for: Co-expression of a SARP Family Activator ChlF2 and a Type II Thioesterase ChlK Led to High Production of Chlorothricin in Streptomyces antibioticus DSM 40725
Source: Front Bioeng Biotechnol. 2020 Aug 21;8:1013. doi: 10.3389/fbioe.2020.01013 (PMC7471628; doi:10.3389/fbioe.2020.01013)
Supplement: Supplementary file 1 [file Data_Sheet_1.docx]

Supplementary Material

# Supplementary Materials and Methods

**Constructions of *S. antibioticus* derivatives**

To construct ΔchlF2, two 1.6-kb DNA fragments flanking the *chlF2* were amplified by PCR using primer pairs F2DML-F/R and F2DMR-F/R, and kanamycin-resistance gene (*kan*) was obtained from pUC119::*neo* with primer pair Bkan*-*F/R. The three PCR products were respectively digested with *Hin*dIII/*Bgl*II, *Bgl*II/*Xba*I and *Bam*HI/*Bgl*II (*Bam*HI and *Bgl*II are [isocaudarner](javascript:;)), and then ligated into *Hin*dIII/*Xba*I-cleaved pKC1139 to generate pLY201. By the homologous recombination on chromosome, *chlF2* was replaced by *kan,* and the resulting mutant ΔchlF2 was further confirmed by Southern blot analysis.

For the complementation of ΔchlF2, a 3.2 kb DNA fragment containing the intact *chlF2*, *chlK* coding sequence and their putative promoter was amplified by PCR with F2zspg-F/R. The PCR product was digested with *Xba*I/*Bam*HI and inserted into the corresponding sites of integrative vector pSET152. The resulting plasmid pSET152::P*_chlK_*-*chlK-chlF2* was introduced into ΔchlF2 by conjugal transfer, generating the complementary strain ΔchlF2/ pSET152::P*_chlK_*-*chlK-chlF2*.

The construction of *chlF2* over-expression strain F2OE was performed based on homologous recombination. Two 2.5-kb DNA fragments corresponding to the upstream and downstream sequence of *chlK* were amplified by PCR amplification using primer pairs F2H1-F/R, F2H2-F/R, kanamycin resistance gene (*kan*) using primer pair Nkan*-*F/R, genome DNA of WT and plasmid pUC119::*neo* as template, respectively. The three PCR products were respectively digested with *Hin*dIII/*Nde*I, *Nde*I/*Xba*I and *Nde*I/*Nde*I, and then inserted into *Hin*dIII/*Xba*I-cleaved pKC1139 to generate pLY202. It was subsequently introduced into WT to generate F2OE, in which *chlK* was replaced by *kan* and its promoter P*_kan_* via homologous recombination.

Plasmid pSET152::P*_chlK_*-*chlK-chlF2* was conjugally transferred into the wild type strain *S. antibioticus* DSM 40725 (WT) and F2OE to generate YL01 and FYL01, respectively, in which the *chlK* and its co-transcribed *chlF2* were driven by the promoter of *chlK* (P*_chlK_*).

To construct YL02 and FYL02, the constitutive promoter P*_kan_* was used to drive the expression of *chlF2*. The 191 bp *kan* promoter sequence was amplified from plasmid pUC119::*neo* by PCR with primers Gkan-F/R. The coding region of *chlF2* was amplified from genomic DNA of WT with primer pair F2P-F/R. Prior to PCR amplification, Gkan-R and F2P-F were phosphorylated with T4 polynucleotide kinase to facilitate subsequent ligation reactions. The *kan* promoter and *chlF2* coding sequence were digested with *Spe*I and *Eco*RI, respectively. *Spe*I-cleaved P*_kan_* promoter, *Eco*RI-cleaved *chlF2* fragment and *Xba*I/*Eco*RI-digested pSET152 (*Spe*I and *Xba*I are [isocaudarner](javascript:;)) were ligated together. The resulting pSET152::P*_kan_*-*chlF2* was individually introduced into WT and F2OE to generate the strains YL02 and FYL02.

To construct YL03 and FYL03, the constitutive promoter P*_kan_* was used to drive the expression of *kan* and *chlF2*. The *kan* promoter coupled with the *kan* and *chlF2* was amplified from genomic DNA of F2OE with primer pair Gkan*-*F/F2P-R. The fragment was obtained after digestion with *Spe*I and *Eco*RI, and ligated into *Xba*I/*Eco*RI-cleaved pSET152 to generate pSET152::P*_kan_*-*kan-chlF2*. The resulting pSET152::P*_kan_*-*kan-chlF2* was individually introduced into WT and F2OE to generate strains YL03 and FYL03, respectively.

The construction of YL04 and FYL04 was performed similarly as mentioned above. The constitutive promoter P*_kan_* was used to drive the expression of *chlK* and *chlF2*. The P*_kan_* promoter was amplified from pUC119::*neo* with primers Gkan-F/R. The coding region of *chlKF2* was amplified from genomic DNA of WT by PCR with primer pair KP-F/F2P-R. Prior to PCR amplification, Gkan-R and KP-F were phosphorylated with T4 polynucleotide kinase to facilitate subsequent ligation reactions. The *kan* promoter fragment and *chlKF2*-coding region were digested with *Spe*I plus *Eco*RI, respectively. The two fragments were ligated together with *Xba*I/*Eco*RI digested pSET152. The resulting pSET152::P*_kan_*-*chlK-chlF2* was individually introduced into WT and F2OE to generate the strains YL04 and FYL04.

For the construction of B4F2OE, two fragments corresponding to 191 bp of the *kan* promoter and 1.3 kb of *chlB4* coding region were obtained by PCR amplification using primer pairs Gkan-F/R and GB4-F/R, plasmid DNA of pUC119::*neo* and genomic DNA of WT as template, respectively. Primers Gkan-R and GB4-F were phosphorylated prior to PCR amplification. The two fragments were then separately digested with *Spe*I and *Xho*I, and ligated into the corresponding sites of the integrative plasmid pIJ10500 to generate pIJ10500::P*_kan_-chlB4*. Subsequently, the resulting plasmid was introduced into the F2OE by conjugal transfer to give the engineered strain B4F2OE (F2OE/ pIJ10500::P*_kan_-chlB4*)*.*

**Southern blot analysis**

Southern blot experiment was performed using a DIG high prime DNA labeling and detection starter kit (Roche) according to the standard protocol (Sambrook, 1989). According to the instructions, genomic DNA of *S. antibioticus* was digested with *Nco*I, separated on 0.8 % agarose gels, and transferred onto nylon membrane. A~1.0 kb kanamycin resistance gene fragment was obtained from pUC119::*neo* and used as a probe.

**Construction of the *gusA*-encoding β-glucuronidase (GUS) reporter system for GUS assays**

To confirm the binding activity of ChlF2, a *gusA*-encoding β-glucuronidase (GUS) reporter system (Sherwood and Bibb, 2013) was used in the heterologous host *S. coelicolor* M1146. The promoters of *chlB4, chlB5, chlC3, chlF1, chlJ, chlK, chlC1, chlE1, chlC6, chlA1, chlM* and *chlL* from *chl* cluster were amplified by PCR using their corresponding primers (see Table S3) and digested with *Nde*I, respectively, while *gusA* amplified from plasmid pGUS was digested with *Xho*I. The promoters along with the *gusA* coding region were ligated into the *Nde*I/*Xho*I-digested pIJ10500 individually to generate the corresponding plasmids. Prior to PCR amplification, all the reverse primers of promoter regions and the forward primer of *gusA* were phosphorylated by T4 polynucleotide kinase. These resulting pIJ10500-derived plasmids were separately integrated into the ΦBT1 (*attB*) site of *S. coelicolor* M1146 via conjugal transfer*.* Exconjugants were selected using hygromycin as resistant marker and confirmed by PCR analysis. These recombinant strains MB4 (*S. coelicolor* M1146/pIJ10500::P*_chlB4_*-*gusA*), MB5 (M1146/pIJ10500::P*_chlB5_*-*gusA*), MC3 (M1146/pIJ10500::P*_chlC3_*-*gusA*), MJ (M1146/pIJ10500::P*_chlJ_*-*gusA*), MF1 (M1146/pIJ10500::P*_chlF1_*-*gusA*), MK (M1146/pIJ10500::P*_chlK_*-*gusA*), MC1 (M1146/pIJ10500::P*_chlC1_*-*gusA*), MC6 (M1146/pIJ10500::P*_chlC6_*-*gusA*), ME1 (M1146/pIJ10500::P*_chlE1_*-*gusA*), MA1 (M1146/pIJ10500::P*_chlA1_*-*gusA*), ML (M1146/pIJ10500::P*_chlL_*-*gusA*) and MM (M1146/pIJ10500::P*_chlM_*-*gusA*) were used as negative controls in β-glucuronidase (GUS) assays and as conjugation recipients. For the subsequent construction, a 2270 bp fragment containing *kan* and *chlF2* driven by P*_kan_* was amplified by PCR from the genomic DNA of F2OE using primer pair F2EX-F/gusF2zspg-R. After digestion with *Eco*RI/*Hin*dIII, the fragment was inserted into the same sites of pKC1139 to generate pKC1139::P*_kan_*-*kan-chlF2*. Then, the resulting plasmid was respectively introduced into above-described *S. coelicolor* M1146 conjugation recipients that contained the corresponding pIJ10500-derived plasmids to obtain M1146 derivatives (FMB4, FMB5, FMC3, FMJ, FMF1, FMK, FMC1, FMC6, FME1, FMA1, FML and FMM). For GUS assays, spores of *S. coelicolor* M1146 derivatives were spread on AS-1 plate containing 5-bromo-4-chloro-3-indolyl-β-D-glucuronide (X-Gluc) at a concentration of 40 μM (Myronovskyi et al., 2011). Chromogenic reaction can be observed after incubation for 4 days at 28 °C. The detection of GUS activity for *S. coelicolor* M1146 derivatives was performed in triplicate.

**Bacterial two-hybrid system**

The bacterial two-hybrid system (BATCH), based on the reconstitution of activity of adenylate cyclase in *E. coli* BTH101 (*cya-*) (Battesti and Bouveret, 2012; Li et al., 2017), was performed to detect the interaction between ChlF2 and ChlK or ChlF2 and kanamycin phosphotransferase (KanP) encoded by *kan*. The *chlF2*, *chlK*, and *kan* fragments were amplified from the genomic DNA of *S. antibioticus* F2OE or WT using primer pairs ThF2-F/R, ThK-F/R and Thka-F/R, respectively. The *Xba*I/*Xho*I-digested *chlF2* coding region was inserted into the same sites of pKT25linker (pEB354) containing T25 domain of adenylate cyclase, and the *Xba*I/*Xho*I-digested *chlK* or *kan* fragment was respectively inserted into *Xba*I/*Xho*I-cleaved pUT18Clinker (pEB355) containing T18 domain of adenylate cyclase, and then the two groups of compatible plasmids (pKT25-*chlF2* and pUT18-*chlK*, pKT25-*chlF2* and pUT18-*kan*) with different replication origins were respectively co-transformed into *E. coli* BTH101 (*cya-*), which is a disruption mutant of coding gene of endogenous adenylate cyclase. The resulting strains were named as SZ-KF2 (BTH101/pKT25-*chlF2* and pUT18-*chlK*) and SZ-kanF2 (BTH101/pKT25-*chlF2* and pUT18-*kan*). The positive control SZ-zip is an *E. coli* BTH101 strain containing pTW1-1 (Wang et al., 2016), in which T25-zip-T18-zip cassette was inserted into plasmid pKT25, and *E. coli* BTH101 containing pUT18Clinker and pKT25linker was designated as a negative control. Once two interesting proteins interacted, the protein-linked T18 domain and T25 domain would then get closer, which could lead to the restoration of adenylate cyclase activity, and activate the transcription of the *lac* operon with the addition of the inducer IPTG. The bacterial suspension was spread on the LB plates supplemented with ampicillin (100 μg/ml), kanamycin (50 μg/ml), X-Gal (40 μg/ml) and IPTG (0.5 mM). The plates were then incubated at 30 °C until a blue coloration appeared.

# Supplementary Figures and Tables

## Supplementary Figures


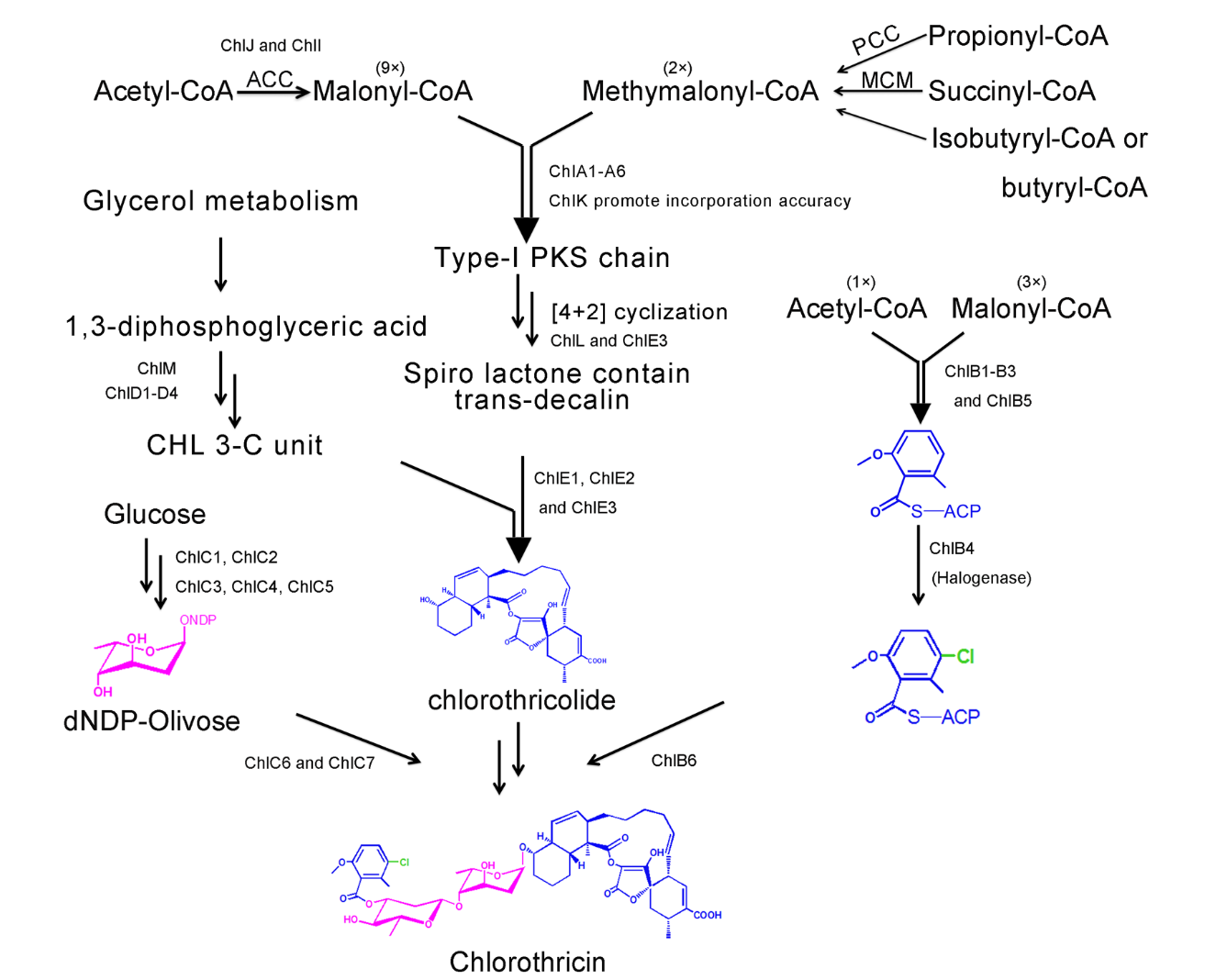


**Figure S1** Diagram of chlorothricin biosynthetic pathways. Blue color denotes the aglycone or 2-methoxy-6-methylsalicylic acid moiety synthesized from type I PKS, purple color denotes sugar moiety, and green color denotes the halogen group. ACC, acetyl-CoA carboxylase; PCC, propionyl-CoA carboxylase; MCM, methylmalonyl-CoA mutase.


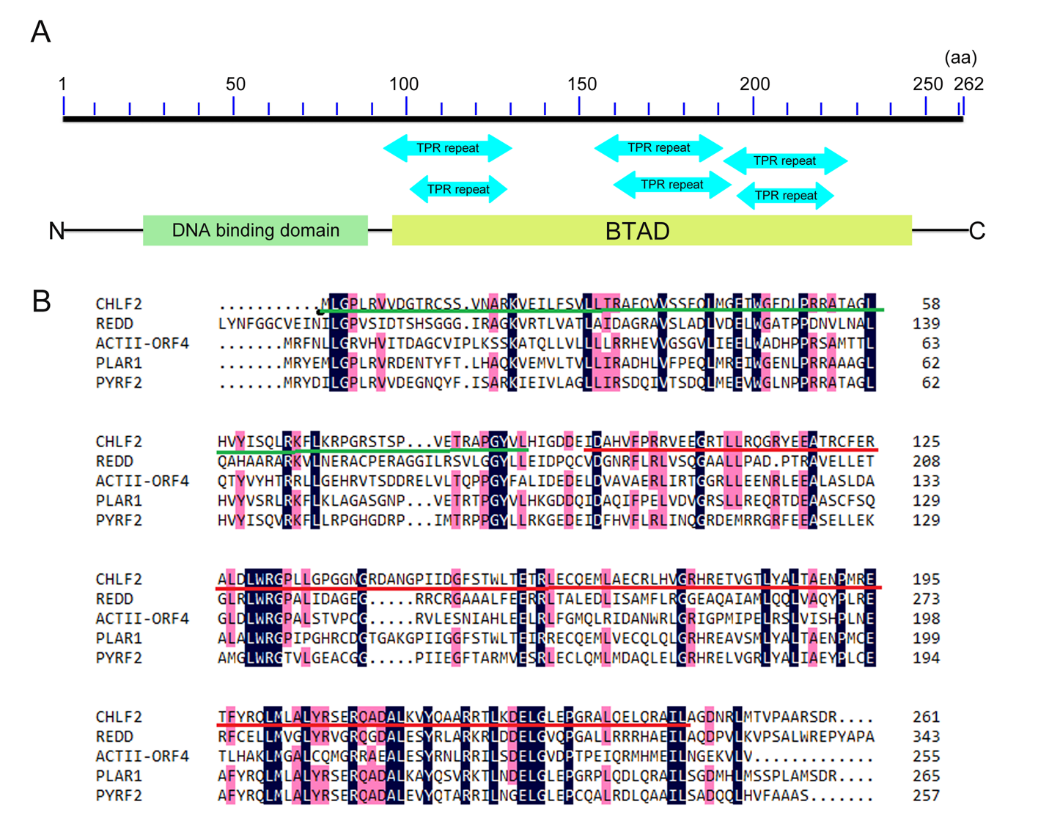


**Figure S2** Domain structure and amino acid alignment of ChlF2 protein. **(A)** Predicted domain structure of ChlF2. The DNA-binding domain and BTAD (bacterial transcriptional activation domain) of ChlF2 are indicated by green or yellow box, respectively. The secondary structure element TPRs (tetratricopeptide repeats) of ChlF2 are indicated by light blue arrow. The full length of protein ChlF2 is 262 aa. **(B)** Amino acid alignment of ChlF2 with selected homologous proteins. Numbers indicate the position of amino acid residues from the N-terminus of the protein. Identical amino acid residues are highlighted in black, and residues with more than 75% similarity are shown in pink. RedD, a transcriptional regulator from *S. coelicolor* A3(2) (Accession number: AAA88556.1); ActII-ORF4, a transcriptional regulator of actinorhodin biosynthesis in *S. coelicolor* A3(2) (AAK32147.1); PlaR1, a transcriptional regulator involved in phenalinolactone biosynthesis in *Streptomyces* sp. Tu6071(CM001165.1); PyrF2, a regulator of pyrroindomycin biosynthesis in *Streptomyces rugosporus* (AFV71314). Green line represents the DNA binding domain, red line represents the BTA domain.


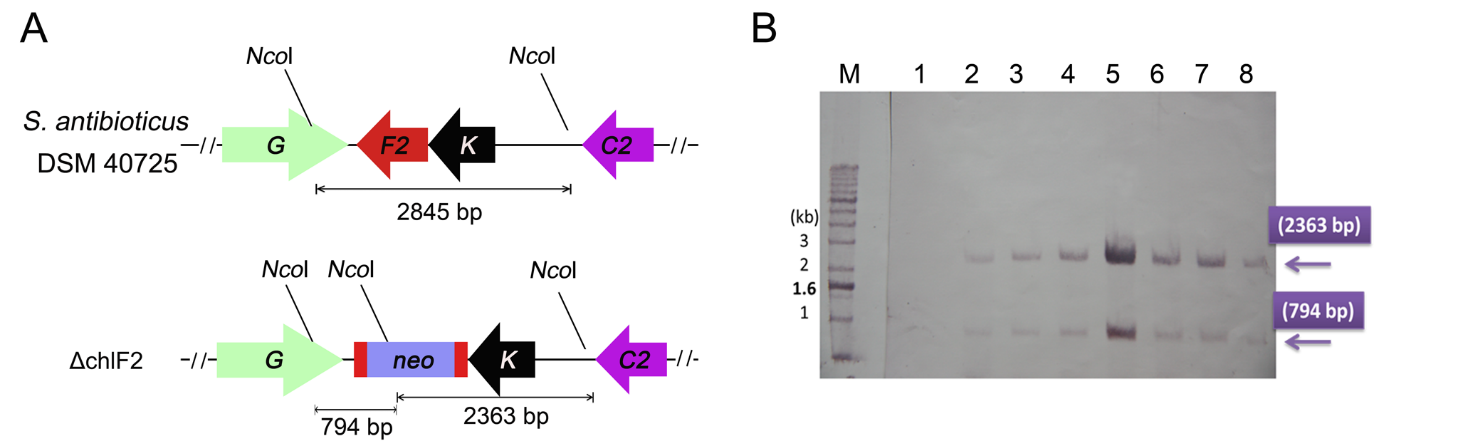


**Figure S3** Southern blot analysis of ΔchlF2. **(A)** Diagram of *Nco*I-digested genomic DNA of *S. antibioticus* DSM40725 and ΔchlF2. *chlF2* was replaced by kanamycin resistance gene *kan (neo)*. The length of DNA fragments digested with *Nco*I is indicated in the picture. **(B)** Southern blot analysis of ΔchlF2. M, marker of DNA fragment size; lane 1, genome DNA of WT, lanes 2-8, genome DNA of ΔchlF2 from different colonies. DNA fragment corresponding to *kan* was labeled as a probe. There is no band appeared in lane 1 due to no *kan* insertion in the chromosome of WT, while two DNA bands corresponding to 794 bp and 2363 bp were observed in lanes 2-8 due to *kan* instead of *chlF2*.


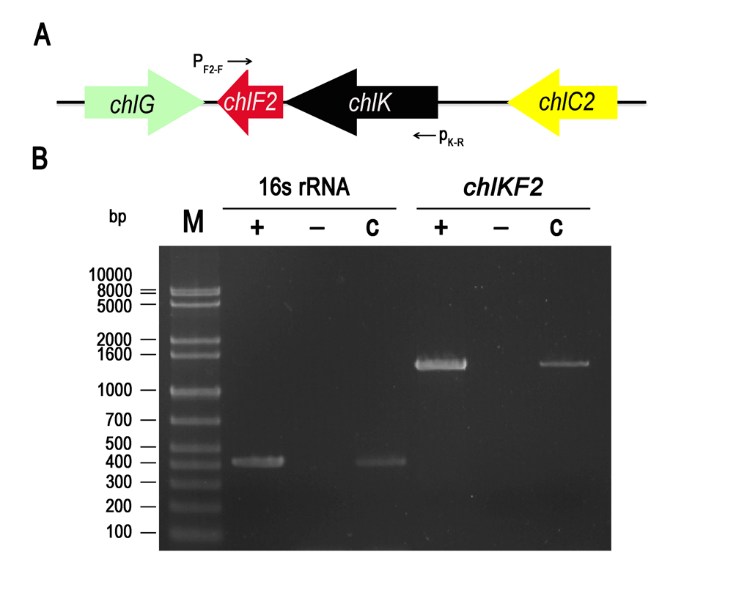


**Figure S4** Co-transcriptional analysis of *chlK* and *chlF2* by RT-PCR. **(A).** Genetic organization of *chlK* and *chlF2*. **(B)**. Co-transcriptional analysis of *chlK* and *chlF2.* Primer pairs PF2-F/PK-R and B16S-F/B16S-R were used to determine the transcriptional profile of *chlKF2* and 16S rRNA coding gene, respectively. M, DNA Ladder; c, cDNA as template; +, genomic DNA as template; -, RNA without reverse transcription as template.





**Figure S5** RT-qPCR transcriptional analysis of *chlF2* in WT and ΔchlF1. WT, *S. antibioticus* DSM 40725, ΔchlF1, *chlF1* disruption strain. The data are presented as the averages of three independent experiments conducted in triplicate. Error bars show standard deviations.


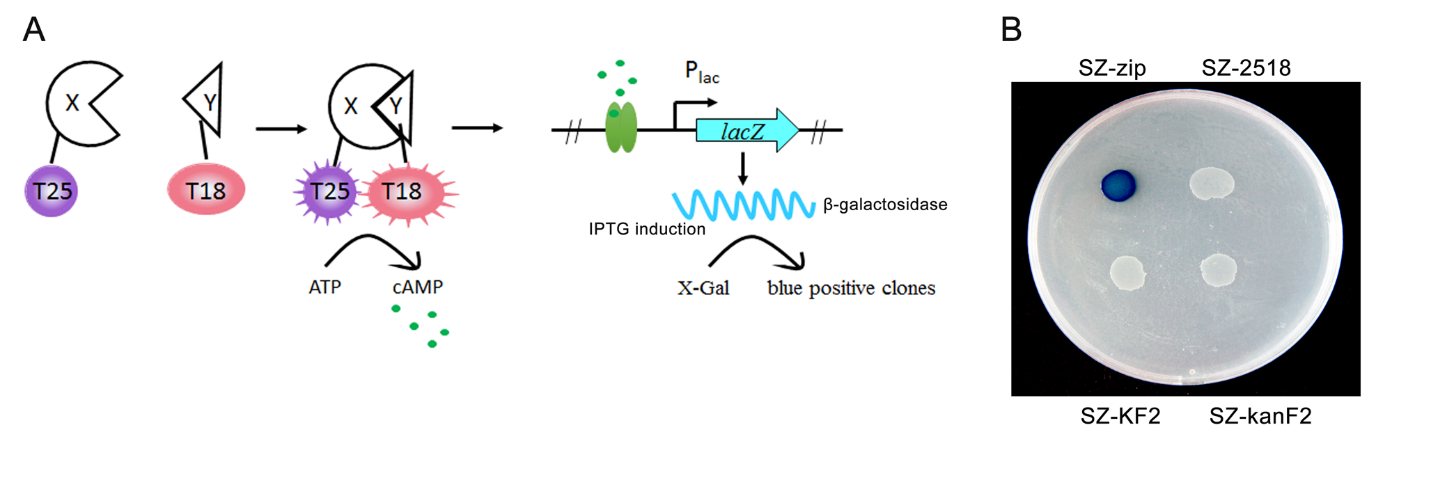


**Figure S6** Protein interaction between ChlF2 and ChlK or KanP. **(A)** Diagram of bacterial two hybrid system assay, X and Y represent the two proteins of interest. Green dots indicate cAMP (cyclic adenosine 3’, 5’-monophosphate). Green ellipse indicates CAP (catabolite activator protein). cAMP/CAP complex binds to the promoter region of *lacZ* and regulates the transcription of *lacZ*. **(B)** The bacterial two hybrid system assay of ChlF2 and ChlK, or ChlF2 and KanP on LB plate containing X-gal. SZ-zip indicates *E. coli* BTH101 harbouring T25-zip-T18-zip cassette used as a positive control; SZ-2518 indicates *E. coli* BTH101 containing the empty vectors pUT18Clinker and pKT25linker used as a negative control; SZ-KF2 indicates *E. coli* BTH101 harbouring pUT18-*chlK* and pKT25-*chlF2*; SZ-kanF2 indicates *E. coli* BTH101 carrying pUT18-*kan* and pKT25-*chlF2*. Blue color indicates the interaction of two proteins, white color indicates no interaction.

## Supplementary Tables

**Table S1 plasmids used in this study**

| Plasmids | Description | Source |
| --- | --- | --- |
| pKC1139 | Apr^R^, *E. coli–Streptomyces* shuttle vector | (Bierman et al., 1992) |
| pSET152 | Apr^R^, *lacZ*, *rep*pMB1**attϕC31*, *oriT* | (Kieser, 2000) |
| pIJ10500 | Hyg^R^, contain *ϕBT1* integrated enzyme gene and integrated site *attP*, derive from *pMS82* | (Pullan et al., 2011) |
| pUC119::*neo* | Amp^R^, derive from pUC119, the DNA template of P*_kan_*-*kan* | This lab |
| pLY201 | A 4.2 kb DNA fragment containing the left and right flanks of *chlF2* and *kan* was inserted into pKC1139, used for *chlF2* deletion. | This study |
| pLY202 | A 6.2 kb DNA fragment containing the left and right flanks of *chlK* and also *kan* plus promoter was inserted into pKC1139, used for *chlF2* high expression | This study |
| pKC1139::P*_kan_*-*kan-chlF2* | pKC1139 contain P*_kan_*-*kan-chlF2,* used for *chlF2* high expression *in S. coelicolor* M1146 | This study |
| pIJ10500::P*_chlB5_-gusA* | pIJ10500 with insertion of *gusA* driven by *chlB5* promoter | This study |
| pIJ10500::P*_chlB4_-gusA* | pIJ10500 with insertion of *gusA* driven by *chlB4* promoter | This study |
| pIJ10500::P*_chlC3_-gusA* | pIJ10500 with insertion of *gusA* driven by *chlC3* promoter | This study |
| pIJ10500::P*_chlJ_-gusA* | pIJ10500 with insertion of *gusA* driven by *chlJ* promoter | This study |
| pIJ10500::P*_chlF1_-gusA* | pIJ10500 with insertion of *gusA* driven by *chlF1* promoter | This study |
| pIJ10500::P*_chlK_-gusA* | pIJ10500 with insertion of *gusA* driven by *chlK* promoter | This study |
| pIJ10500::P*_chlC1_-gusA* | pIJ10500 with insertion of *gusA* driven by *chlC1* promoter | This study |
| pIJ10500::P*_chlC6_-gusA* | pIJ10500 with insertion of *gusA* driven by *chlC6* promoter | This study |
| pIJ10500::P*_chlE1_-gusA* | pIJ10500 with insertion of *gusA* driven by *chlE1* promoter | This study |
| pIJ10500::P*_chlA1_-gusA* | pIJ10500 with insertion of *gusA* drived by *chlA1* promoter | This study |
| pIJ10500::P*_chlM_-gusA* | pIJ10500 with insertion of *gusA* driven by *chlM* promoter | This study |
| pIJ10500::P*_chlL_-gusA* | pIJ10500 with insertion of *gusA* driven by *chlL* promoter | This study |
| pSET152::P*_chlK_*-*chlK-chlF2* | pSET152 containing the intact *chlK* and *chlF2* driven by their native promoter P*_chlK_* | This study |
| pSET152::P*_kan_-chlF2* | pSET152 containing *chlF2* driven by P*_kan_* promoter | This study |
| pSET152::P*_kan_*-*chlK-chlF2* | pSET152 containing *chlK* and *chlF2* driven by P*_kan_* promoter | This study |
| pSET152::P*_kan_*-*kan-chlF2* | pSET152 containing *kan* and *chlF2* driven by P*_kan_* promoter | This study |
| pIJ10500::*P_kan_-chlB4* | pIJ10500 with insertion of *chlB4* driven by *P_kan_* promoter | This study |
| pUT18Clinker (pEB355) | Amp^R^, carrying the sequence for the T18 domain of adenylate cyclase, ColE1 origin | (Battesti and Bouveret, 2012) |
| pKT25linker (pEB354) | Kan^R^, carrying the sequence for the T25 domain of adenylate cyclase, p15A origin | (Battesti and Bouveret, 2012) |
| pTW1-1 | Kan^R^, p15A origin, derived from pKT25, containing T25-zip and T18-zip | (Wang et al., 2016) |
| pKT25-*chlF2* | Kan^R^，derived from pKT25Clinker, containing T25 domain and *chlF2* | This study |
| pUT18-*chlK* | Amp^R^, derived from pUT18Clinker, containing T18 domain and *chlK* | This study |
| pUT18-*kan* | Amp^R^, derived from pUT18Clinker, containing T18 domain and *kan* | This study |

**Table S2 Strains used in this study.**

| Strains | Descriptions | Source |
| --- | --- | --- |
| ***Streptomyces antibioticus*** | | |
| DSM 40725 | Wild type (WT), chlorothricin producer | (Jia et al., 2006) |
| ΔchlF2 | *chlF2* disruption mutant | This study |
| ΔchlF2/pSET152::P*_chlK_-chlK-chlF2* | *chlF2* complementary strain | This study |
| ΔchlF2/pSET152::P*_upchlF2_*-*chlF2* | *chlF2* expressed in ΔchlF2 under the control of a short stretch of upstream *chlF2* coding sequence | This study |
| F2OE | *chlF2* over-expression strain, *chlK* was replaced by *kan* and P*_kan_* promoter, *chlF2* co-transcribed with *kan* under the control of P*_kan_* | This study |
| YL01 | WT containing pSET152::P*_chlK_-chlK-chlF2* | This study |
| YL02 | WT containing pSET152::P*_kan_-chlF2* | This study |
| YL03 | WT containing pSET152::P*_kan_-kan-chlF2* | This study |
| YL04 | WT containing pSET152::P*_kan_-chlK-chlF2* | This study |
| FYL01 | F2OE containing pSET152::P*_chlK_-chlK-chlF2* | This study |
| FYL02 | F2OE containing pSET152:: P*_kan_-chlF2* | This study |
| FYL03 | F2OE containing pSET152::P*_kan_ -kan-chlF2* | This study |
| FYL04 | F2OE containing pSET152::P*_kan_-chlK-chlF2* | This study |
| B4F2OE | F2OE containing pIJ10500::P*_kan_-chlB4* | This study |
| ***Streptomyces coelicolor*** |  |  |
| M1146 | *Δact Δred Δcpk Δcda* | (Gomez-Escribano and Bibb, 2011) |
| M1146/pKC1139 | M1146 containing pKC1139 | This study |
| MB5 | M1146 containing pIJ10500::P*_chlB5_-gusA* | This study |
| MB4 | M1146 containing pIJ10500::P*_chlB4_-gusA* | This study |
| MC3 | M1146 containing pIJ10500::P*_chlC3_-gusA* | This study |
| MJ | M1146 containing pIJ10500::P*_chlJ_-gusA* | This study |
| MF1 | M1146 containing pIJ10500::P*_chlF1_-gusA* | This study |
| MK | M1146 containing pIJ10500::P*_chlK_-gusA* | This study |
| MC1 | M1146 containing pIJ10500::P*_chlC1_-gusA* | This study |
| MC6 | M1146 containing pIJ10500::P*_chlC6_-gusA* | This study |
| ME1 | M1146 containing pIJ10500::P*_chlE1_-gusA* | This study |
| MA1 | M1146 containing pIJ10500::P*_chlA1_-gusA* | This study |
| MM | M1146 containing pIJ10500::P*_chlM_-gusA* | This study |
| ML | M1146 containing pIJ10500::P*_chlL_-gusA* | This study |
| FMB5 | M1146 containing pKC1139::P*_kan_*-*kan-chlF2* and pIJ10500::P*_chlB5_-gusA* | This study |
| FMB4 | M1146 containing pKC1139::P*_kan_*-*kan-chlF2* and pIJ10500::P*_chlB4_-gusA* | This study |
| FMC3 | M1146 containing pKC1139::P*_kan_*-*kan-chlF2* and pIJ10500::P*_chlC3_-gusA* | This study |
| FMJ | M1146 containing pKC1139::P*_kan_*-*kan-chlF2* and pIJ10500::P*_chlJ_-gusA* | This study |
| FMF1 | M1146 containing pKC1139::P*_kan_*-*kan-chlF2* and pIJ10500::P*_chlF1_-gusA* | This study |
| FMK | M1146 containing pKC1139::P*_kan_*-*kan-chlF2* and pIJ10500::P*_chlK_-gusA* | This study |
| FMC1 | M1146 containing pKC1139::P*_kan_*-*kan-chlF2* and pIJ10500::P*_chlC1_-gusA* | This study |
| FMC6 | M1146 containing pKC1139::P*_kan_*-*kan-chlF2* and pIJ10500::P*_chlC6_-gusA* | This study |
| FME1 | M1146 containing pKC1139::P*_kan_*-*kan-chlF2* and pIJ10500::P*_chlE1_-gusA* | This study |
| FMA1 | M1146 containing pKC1139::P*_kan_*-*kan-chlF2* and pIJ10500::P*_chlA1_-gusA* | This study |
| FMM | M1146 containing pKC1139::P*_kan_*-*kan-chlF2* and pIJ10500::P*_chlM_-gusA* | This study |
| FML | M1146 containing pKC1139::P*_kan_*-*kan-chlF2* and pIJ10500::P*_chlL_-gusA* | This study |
| ***Eschericia coli*** | | |
| JM109 | *recA1*, *endA1*, *gyrA96*, *thi*-*1*, *hsdR17*, *supE44*, *relA1*, Δ(lac-proAB)/F’  [traD36, proAB + lacIq, lacZΔM15] | Invitrogen |
| ET12567/pUZ8002 | *dam*− *dcm*− *hsdM*− pUZ8002 | (Kieser, 2000) |
| BTH101 | *F’, cya-99, araD139, galE15, galK16, rpsL1 (Str^R^), hsdR2, mcrA1, mcrB1, relA1* | (Battesti and Bouveret, 2012) |
| SZ-zip | BTH101 containing pTW1-1 with T25-zip-T18-zip, the positive control | (Wang et al., 2016) |
| SZ-2518 | BTH101 containing pKT25linker (pEB354) and pUT18Clinker (pEB355), the negative control | This study |
| SZ-KF2 | BTH101 containing pKT25-*chlF2* and pUT18-*chlK* | This study |
| SZ-kanF2 | BTH101 containing pKT25-*chlF2* and pUT18-*kan* | This study |
| **Indicator strains** |  |  |
| *Bacillus subtilis* CGMCC1.1630 | Indicator strain for MIC | CGMCC |
| *Bacillus cereus* CGMCC1.1626 | Indicator strain for MIC | CGMCC |
| *Staphylococcus aureus* CGMCC1.89 | Indicator strain for MIC | CGMCC |
| *Staphylococcus epidermidis* ATCC 35984 | Indicator strain for MIC | (Lu et al., 2015) |
| *Streptococcus pyogenes* #2 | Indicator strain for MIC | (Lu et al., 2015) |
| *Streptococcus pneumoniae* 010 | Indicator strain for MIC | (Lu et al., 2015) |
| *Pseudomonas aeruginosa* PA14 | Indicator strain for MIC | (Lu et al., 2015) |
| *Candida albicans* CGMCC 2.4159 | Indicator strain for MIC | CGMCC |
| *Alternaria longipes* CGMCC 3.2946 | Indicator strain for MIC | CGMCC |
| **Cell lines** |  |  |
| Human lung carcinoma cell line A549 | Bioassays for anticancer activity | * |
| human lung adenocarcinoma cell line Calu-3 | Bioassays for anticancer activity | * |
| human hepatocellular  carcinoma cell line HepG2 | Bioassays for anticancer activity | * |
| human breast adenocarcinoma cell line MCF-7 | Bioassays for anticancer activity | * |

Asterisk indicates cell lines were purchased from the Cell Bank of Chinese Academy

of Sciences, Shanghai, China

**Table S3 Primers used in this study**

| Name | Sequence(5’ to 3’) | Purpose |
| --- | --- | --- |
| F2DML-F | CATGAAGCTTTTCGACACCACCCTGCTCACCATC | *chlF2* deletion |
| F2DML-R | CATGAGATCTTCCGACCGCTGACCGCCCCACCGC | *chlF2* deletion |
| F2DMR-F | CATGAGATCTATCTCCACTTTGCGGGCATTCACAG | *chlF2* deletion |
| F2DMR-R | CATGTCTAGATTCCGTGGCTTGTCCAGCGATACCG | *chlF2* deletion |
| Bkan-F | CATGGGATCCGCATGCCTGCAGGTCGACTCT | *chlF2* deletion |
| Bkan-R | CATGAGATCTGAGCTCGGTACCCGAACCCCAG | *chlF2* deletion |
| F2zspg-F | CATGTCTAGAAGATCTACAACATCGGTGCCGGAGA | *chlF2* complementation |
| F2zspg-R | CGGGATCCCGCGGTGCCTACGGCATCACCTTCTGGT | *chlF2* complementation |
| F2H1-F | CCCAAGCTTATGAGCGCTTCCACGCAAGTCAACGAGAC | *chlF2* over-expression |
| F2H1-R | GGAATTCCATATGTGGTCACTTCTACCTGGACGTCCACC | *chlF2* over-expression |
| F2H2-F | GGAATTCCATATGACACTGTCGACGAATGGCTCCCTGCG | *chF2* over-expression |
| F2H2-R | GCTCTAGAAGTGGCTGGTGAGCCACGACCGTGAGGT | *chlF2* over-expression |
| Nkan-F | GGAATTCCATATGGCATGCCTGCAGGTCGACTCT | *chlF2* over-expression |
| Nkan-R | GGAATTCCATATGGAGCTCGGTACCCGAACCCCAG | *chlF2* over-expression |
| B16S-F | GGTGTTGGCGACATTCCACGT | co-transcription analysis |
| B16S-R | GTACCGGCCATTGTAGCACGT | co-transcription analysis |
| PF2-F | TGATAGACCTTCAGCGCGTCGGCCT | co-transcription analysis |
| PK-R | TCCGGCTCGTGTGTTTCC | co-transcription analysis |
| F2EX-F | CGGAATTCCATGCCTGCAGGTCGACTCTAG | *gusA* reporter system |
| gusF2zspg-R | CCCAAGCTTCGGTGCCTACGGCATCACCTTCTGGT | *gusA* reporter system |
| gusA-F | ATGACCGGTCTGCGGCCCGTCG | *gusA* PCR amplification |
| gusA-R | CCTCGAGTCACTGCTTCCCGCCCTGCTGCGGC | *gusA* PCR amplification |
| gus-B5F | GGAATTCCATATGAGCTCGTGCAACTCGGCGTCCT | *chlB5* promoter for GUS assay |
| gus-B5R | CCTGGGGTCCTGGAGTGCTGCGGAAGTA | *chlB5* promoter for GUS assay |
| gus-B4F | GGAATTCCATATGTGCGCCGTCGTGAAGGTGCTCA | *chlB4* promoter for GUS assay |
| gus-B4R | ACTTCCTCCTGGTCGTCGTTCAGCCGGC | *chlB4* promoter for GUS assay |
| gus-C3F | GGAATTCCATATGTCAAGGAGTACAGGGCCGAACT | *chlC3* promoter for GUS assay |
| gus-C3R | CCAGAGCCTCCTGAAGGGCCTGCCCGGA | *chlC3* promoter for GUS assay |
| gus-JF | GGAATTCCATATGTCAGCGCCATCGCCATGCCTTC | *chlJ* promoter for GUS assay |
| gus-JR | GCACCTCATCGTTGACACCGGGTCTTAA | *chlJ* promoter for GUS assay |
| gus-F1F | GGAATTCCATATGAATCCCGTGCAGTTCGGCGGTA | *chlF1* promoter for GUS assay |
| gus-F1R | GTAGTAAATGCTTACAGAGATCCGCCCG | *chlF1* promoter for GUS assay |
| gus-KF | GGAATTCCATATGGCGAGATCTACAACATCGGTGC | *chlK* promoter for GUS assay |
| gus-KR | CTCGTATCACTCCGTCAGAAGCGCATGC | *chlK* promoter for GUS assay |
| gus-C1F | GGAATTCCATATGAGCTGCGCAACGCCGAGACCGT | *chlC1* promoter for GUS assay |
| gus-C1R | CGGGATCACTCACACCAATCCTTCGAAG | *chlC1* promoter for GUS assay |
| gus-C6F | GGAATTCCATATGGTAGTCCAGCAGTTGGACGATC | *chlC6* promoter for GUS assay |
| gus-C6R | GCGCATGGGTTACTCCGTGTCCCCGCCG | *chlC6* promoter for GUS assay |
| gus-E1F | GGAATTCCATATGTGCAGACCGGCATGCCGGATCG | *chlE1* promoter for GUS assay |
| gus-E1R | CGGGGTCTTCCGCTCTCTGTCGGGGCTG | *chlE1* promoter for GUS assay |
| gus-A1F | GGAATTCCATATGGTGAACCTGGGCTCTGGAAGCTGG | *chlA1* promoter for GUS assay |
| gus-A1R | ATCCTGATCGTTCTCACACGCTCACTGA | *chlA1* promoter for GUS assay |
| gus-MF | GGAATTCCATATGTCACGCCATCGCGAACGACGTC | *chlM* promoter for GUS assay |
| gus-MR | GCCGCCCATCCTCGGCGCGGACGCTAAAG | *chlM* promoter for GUS assay |
| gus-LF | GGAATTCCATATGAGGCTGTAGCGGATGTCACCGG | *chlL* promoter for GUS assay |
| gus-LR | GGTCGTCTCACTGTCAGGCACTCGGCCG | *chlL* promoter for GUS assay |
| q16S rRNA-F | ACATACACCGGAAAGCATTAGA | Q-PCR normalization |
| q16S rRNA-R | TAACCCAACATCTCACGACAC | Q-PCR normalization |
| qC3-F | CATAGAACCCGACGATGGAC | *chlC3* qPCR |
| qC3-R | CTTGACCAGGATACCGAGAAC | *chlC3* qPCR |
| qC6-F | GAACCGGGTGACCAACTG | *chlC6* qPCR |
| qC6-R | CGCTGTACGAGCGGTTC | *chlC6* qPCR |
| qB4-F | GTGTTCGAGAGCGAGATGTT | *chlB4* qPCR |
| qB4-R | CCGTATTTCTTCGGGAAGCC | *chlB4* qPCR |
| qE1-F | GATGCTGGCCTACGAACTC | *chlE1* qPCR |
| qE1-R | CAGCAGTTGGACGATCCG | *chlE1* qPCR |
| qM-F | CGATGGAGGAACCGATGAG | *chlM* qPCR |
| qM-R | TTGGGCATCCCGAAGTG | *chlM* qPCR |
| qL-F | AGAACCCGTCCTGGTACTC | *chlL* qPCR |
| qL-R | AGGAGGCGCTGAAGAAATG | *chlL* qPCR |
| qJ-F | GTCCATCAGCTTGTGGATCTT | *chlJ* qPCR |
| qJ-R | CCGGACGGTCTTCGTCTA | *chlJ* qPCR |
| qF2-F | GACGATGAGGTACGAAATGCT | *chlF2* qPCR |
| qF2-R | TTGCGGGCATTCACAGAT | *chlF2* qPCR |
| Gkan-F | GACTAGTCGCATGCCTGCAGGTCGACTCT | *kan* promoter amplification |
| Gkan-R | GCGAAACGATCCTCATCCTGTCT | *kan* promoter amplification |
| F2P-F | ATGCTTGGTCCGCTCCGCGTCGTGGA | *chlF2 over-expression* |
| KP-F | TTGTCTCCTACTCCCGACGCGACGGA | *chlKF2 over-expression* |
| F2P-R | CCGGAATTCTCAGCGGTCGGAGCGGGCTGCGG | *chlF2* or *chlKF2 over-expression* |
| GB4-F | ATGCAGCCCGATTTCGACGCCG | *chlB4* PCR amplification |
| GB4-R | CCGCTCGAGTCAGAACGTGGGTGCTGCCGA | *chlB4* PCR amplification |
| ThF2-F | GCTCTAGATATGCTTGGTCCGCTCCGCGT | Two-hybrid system |
| ThF2-R | CCGCTCGAGTCAGCGGTCGGAGCGGGCTG | Two-hybrid system |
| ThK-F | GCTCTAGATTTGTCTCCTACTCCCGACGC | Two-hybrid system |
| ThK-R | CCGCTCGAGTCATCGTCCGGTTGCCTCCAA | Two-hybrid system |
| Thka-F | GCTCTAGATATGATTGAACAAGATGGATT | Two-hybrid system |
| Thka-R | CCGCTCGAGTCAGAAGAACTCGTCAAGAA | Two-hybrid system |

## Supplementary References

Battesti, A., and Bouveret, E. (2012). The bacterial two-hybrid system based on adenylate cyclase reconstitution in *Escherichia coli*. *Methods* 58(4)**,** 325-334. doi: 10.1016/j.ymeth.2012.07.018.

Bierman, M., Logan, R., O'Brien, K., Seno, E.T., Rao, R.N., and Schoner, B.E. (1992). Plasmid cloning vectors for the conjugal transfer of DNA from *Escherichia coli* to *Streptomyces spp*. *Gene* 116(1)**,** 43-49. doi: 10.1016/0378-1119(92)90627-2.

Gomez-Escribano, J.P., and Bibb, M.J. (2011). Engineering *Streptomyces coelicolor* for heterologous expression of secondary metabolite gene clusters. *Microb Biotechnol* 4(2)**,** 207-215. doi: 10.1111/j.1751-7915.2010.00219.x.

Jia, X.Y., Tian, Z.H., Shao, L., Qu, X.D., Zhao, Q.F., Tang, J., et al. (2006). Genetic characterization of the chlorothricin gene cluster as a model for spirotetronate antibiotic biosynthesis. *Chem Biol* 13(6)**,** 575-585. doi: 10.1016/j.chembiol.2006.03.008.

Kieser, T., Bibb, M.J., Buttner, M.J., Chater, K.F., and Hopwood, D.A . (2000). Practical *Streptomyces* Genetics. *The John Innes Foundation, Norwich*.

Li, X., Wang, J., Shi, M., Wang, W., Corre, C., and Yang, K. (2017). Evidence for the formation of ScbR/ScbR2 heterodimers and identification of one of the regulatory targets in *Streptomyces coelicolor*. *Appl Microbiol Biotechnol* 101(13)**,** 5333-5340. doi: 10.1007/s00253-017-8275-8.

Lu, C., Liao, G., Zhang, J., and Tan, H. (2015). Identification of novel tylosin analogues generated by a *wblA* disruption mutant of *Streptomyces ansochromogenes*. *Microb Cell Fact* 14**,** 173. doi: 10.1186/s12934-015-0359-5.

Myronovskyi, M., Welle, E., Fedorenko, V., and Luzhetskyy, A. (2011). Beta-glucuronidase as a sensitive and versatile reporter in *actinomycetes*. *Appl Environ Microbiol* 77(15)**,** 5370-5383. doi: 10.1128/AEM.00434-11.

Pullan, S.T., Chandra, G., Bibb, M.J., and Merrick, M. (2011). Genome-wide analysis of the role of GlnR in *Streptomyces venezuelae* provides new insights into global nitrogen regulation in actinomycetes. *BMC Genomics* 12(1)**,** 175. doi: 10.1186/1471-2164-12-175.

Sambrook, J., Fritsch, E. F., and Maniatis, T. (1989). Molecular Cloning: A Laboratory Manual, 2nd Ed.,. *Cold Spring Harbor Laboratory Press, Cold Spring Harbor, NY.*

Sherwood, E.J., and Bibb, M.J. (2013). The antibiotic planosporicin coordinates its own production in the actinomycete *Planomonospora alba*. *Proc Natl Acad Sci U S A* 110(27)**,** E2500-2509. doi: 10.1073/pnas.1305392110.

Wang, J., Wang, W., Zhao, H., and Yang, K. (2016). The modified bacterial two-hybrid system. *Sheng Wu Gong Cheng Xue Bao* 32(2)**,** 231-240.
